# The Échancrure of the Uncovertebral Joint: A Forgotten Structure of the C3-C7 Cervical Vertebral Bodies

**DOI:** 10.7759/cureus.32471

**Published:** 2022-12-13

**Authors:** Matthew Protas, Juan J Cardona, Arada Chaiyamoon, David Ezra, Ryan M. Glynn, Sassan Keshavarzi, Joe Iwanaga, Aaron S Dumont, R. Shane Tubbs

**Affiliations:** 1 Department of Neurosurgery, State University of New York Upstate Medical University, Syracuse, USA; 2 Department of Neurosurgery, Tulane University School of Medicine, New Orleans, USA; 3 Department of Anatomy, Khon Kaen University, Khon Kaen, THA; 4 Department of Anatomy and Anthropology, Tel Aviv University, Tel Aviv, ISR; 5 Department of Anatomy and Physiology, Tel Aviv Yaffo Academic College, Yaffo, ISR; 6 Department of Neurology, Tulane University School of Medicine, New Orleans, USA; 7 Department of Anatomical Sciences, St. George's University, True Blue, GRD; 8 Department of Structural and Cellular Biology, Tulane University School of Medicine, New Orleans, USA; 9 Department of Neurosurgery and Neuroscience, Ochsner Health System, New Orleans, USA

**Keywords:** osteological study, cervical surgical approaches, surgical anatomy, échancrure, uncinate process, cervical spine

## Abstract

Introduction

The *échancrure* (a French term meaning "indentation") of the cervical vertebrae is the poorly defined articular part on the inferolateral aspect of the cervical spine body, which, with the uncinate processes of the associated caudal vertebra, makes up the joints of Luschka (uncovertebral joint). With no known previous studies on the *échancrure*, the present anatomical study aimed to better elucidate this structure, its prevalence, and its relationships to the adjacent intervertebral foramen and uncinate process.

Methods

We observed 50 adult cervical spines (100 sides) for the presence of an *énchancrure*. When an *énchancrure* was identified, its morphometry was documented and photographed. Measurements included the width and height of the *énchancrure*. The relationship with the adjacent uncinate process was also studied. Any correlation between the size and shape of the adjacent uncinate process and the *énchancrure* was recorded.

Results

An*énchancrure* was found at all levels of the cervical vertebrae except at C1 and C7 and was clearly visible on 88% of the sides. The *énchancrure*, more or less, conformed to the reciprocal shape of the uncinate process, which was found on all sides. The shapes were roughly arched, ovoid, or linear. These structures were always in an anterolateral position on the body of the vertebra and just outside the apophyseal ring. The mean height of the *énchancrure* was 2.1 mm. The length of the uncinate process correlated positively (r=0.8) to the size of the adjacent *énchancrure*. The height of the *énchancrure* was inversely related to the diameter of the adjacent intervertebral foramen. The mean width was 8.3 mm. These structures tended to be largest at C3 and C4 vertebral levels and were smallest at C5 and C6 levels. The *énchancrure* was most in contact with the uncinate process with lateral flexion of the cervical spine and in specimens with a longer uncinate process, e.g., C6. The *énchancrure* was also found to be wider in cases of cervical spine degeneration involving the body of the cervical vertebrae. Degeneration of the uncovertebral joint was most often seen at the *énchancrure* and not at the adjacent uncinate process.

Conclusions

We found that the *énchancrure* is found in the majority of cervical spines. These structures tended to be largest at C3 and C4 vertebral levels and were smallest at C5 and C6 levels, and they had more prominence when the adjacent uncinate process was enlarged. The *énchancrure* should be considered a normal feature of the inferolateral aspect of the cervical vertebrae. Future clinical studies are necessary to better elucidate their functional significance.

## Introduction

In 1858, von Luschka first described the uncovertebral joints of the cervical spine [1]. He considered these homologs of the costovertebral articulations. The joints of Luschka (uncovertebral joints) are most frequently present on the C3-C7 vertebrae [2]. These joints are composed of two parts: the well-known inferior part, made up of an upward projection called the uncinate process, and the nearly forgotten downward receptacle termed by the French the échancrure (indentation) and in Latin, the incus (anvil) [1,3]. The uncinate process has been described as having three types. The type I process does not encroach on the adjacent intervertebral foramen; the type II uncinate process projects in a superior posterolateral direction to decrease the intervertebral foraminal diameter; the type III process lacks an incline of projection but is sufficiently high-riding to create foraminal encroachment [4]. Unlike the uncinate process, the échancrure part of the joint of Luschka is poorly described in the literature and, to our knowledge, has yet to be examined via anatomical studies. Therefore, the aim of this osteological study was to improve our knowledge of the échancrure and better elucidate its contributions and relationships to the joints of Luschka, adjacent intervertebral foramina, and the uncinate process.

## Materials and methods

We observed 50 adult cervical spines (100 sides) for the presence of an énchancrure. The specimens were skeletal material from our university's medical school teaching collection. The collection was derived primarily from North American skeletons. As the majority of these vertebrae were not attached to the remaining skeleton, the exact age and gender could not be determined. When an énchancrure was identified, its morphometry was documented and photographed. Measurements included the width and height of the énchancrure. The relationship with the adjacent uncinate process was also studied. Any correlation between the size and shape of the adjacent uncinate process and the énchancrure was analyzed (Pearson’s correlation coefficient) and recorded. All measurements were made using microcalipers (Mitutoyo, Japan). A surgical microscope (Zeiss, Germany) was used for magnification. Statistical analysis was performed (Student's t-test) between the left and right sides and vertebral levels C2 to C7, and significance was set at p<0.05. The authors state that every effort was made to follow all local and international ethical guidelines and laws that pertain to the use of human cadaveric donors in anatomical research [5].

## Results

**Figure 1:**
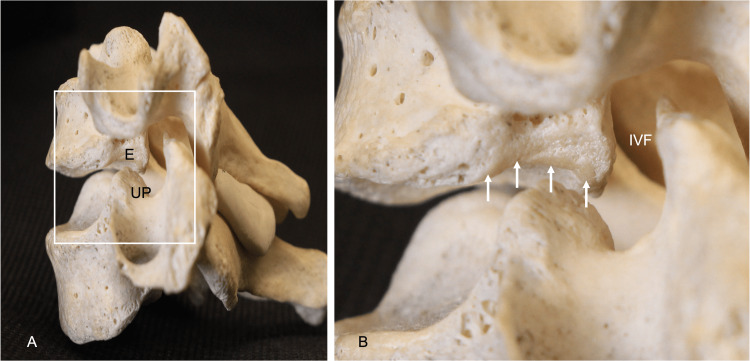
(A): Left lateral view of the énchancrure (E) and uncinate process (UP). (B): The magnified view shows the énchancrure (arrows) and the relationship to the adjacent intervertebral foramen (IVF).

**Figure 2:**
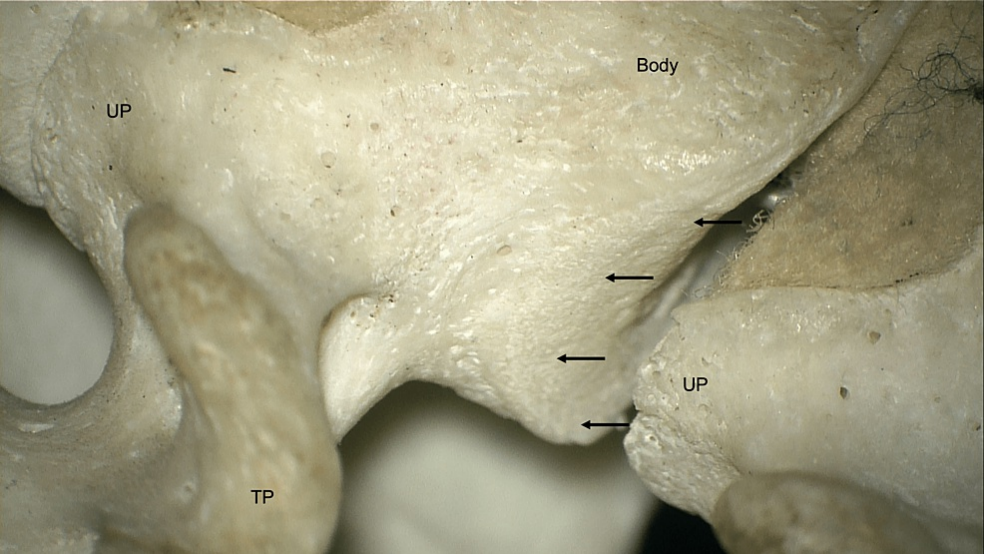
Anterolateral view of an enlarged right énchancrure (arrows) and adjacent uncinate process (UP). For reference, note the ipsilateral transverse process (TP).

**Figure 3:**
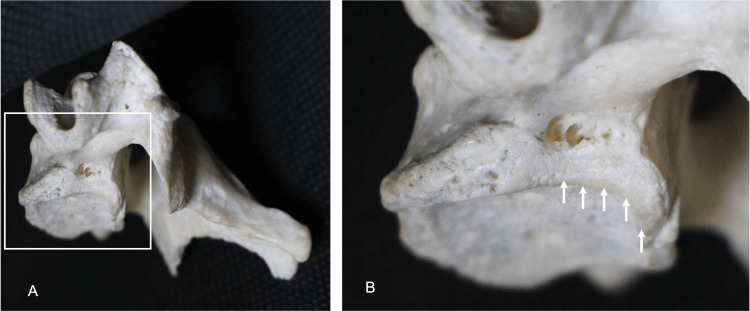
(A): Left view of the énchancrure. (B): The magnified view shows the énchancrure (arrows) with degeneration of the vertebral body.

**Figure 4:**
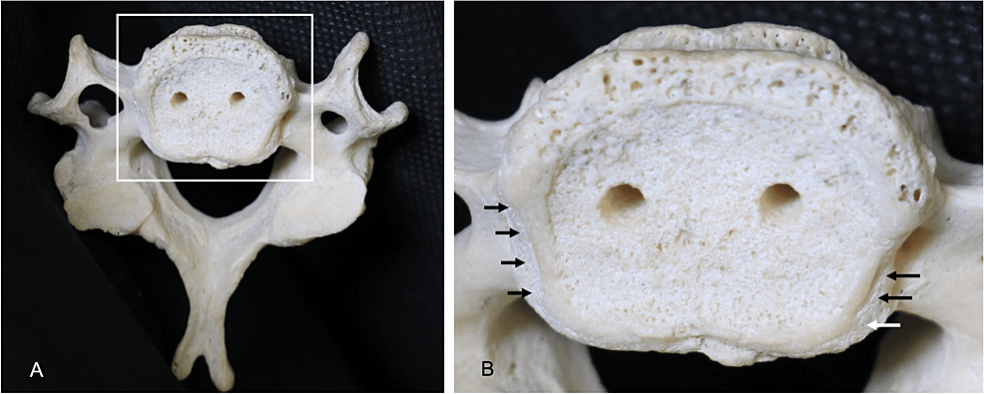
(A): Inferior view of the C6 vertebra. (B): The magnified view shows the C6 vertebra, noting the location of the énchancrures (arrows) outside of the apophyseal ring. Note that the holes in the vertebral bodies are artifacts due to the wiring used to link the vertebrae.

**Figure 5:**
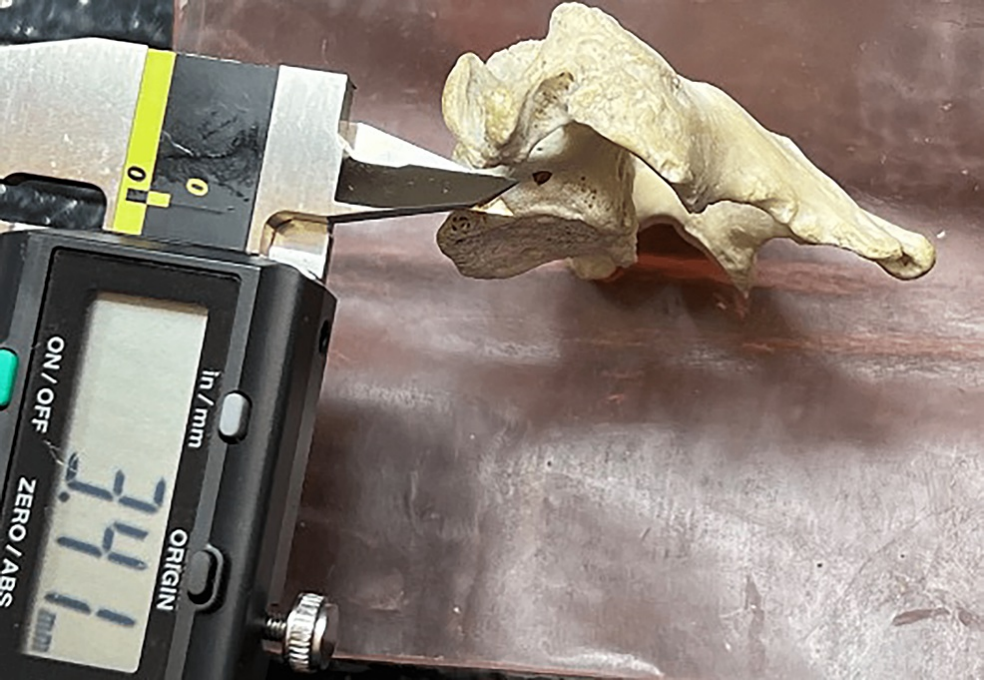
Microcalipers being used to measure the énchancrure.

An énchancrure was found at all levels of the cervical vertebrae except C1 and was clearly visible on 88% of sides. As T1 often did not have an uncinate process, a C7 énchancrure was uncommon (<20%). The énchancrure, more or less, conformed to the reciprocal shape of the uncinate process, which was found on all sides. However, in some cases, the width of the énchancrure was up to 3 mm wider than the reciprocating part of the uncinate process. The shapes were roughly arched, ovoid, or linear (Figures 1-5). These structures were always in an anterolateral position on the body of the vertebra and just outside the apophyseal ring. 

The height of the énchancrure ranged from 0.8 to 4.3 mm (mean 2.1 mm). The length of the uncinate process correlated positively (r=0.8) to the size of the adjacent énchancrure. The height of the énchancrure was inversely related to the diameter of the adjacent intervertebral foramen. The mean width was 8.3 mm (range 6.4 to 9.9 mm). There were no statistically significant differences identified between the sides for these measurements (Table 1).

**Table 1:** Summary of morphometric measurements of the énchancrure.

Variables	Range	Mean	p-value
Height	0.8-4.3 mm	2.1 mm	>0.05
Width	6.4-9.9 mm	8.3 mm	>0.05
Presence at the vertebral level	C1	0%	
	C2-C7	88 sides (88%)	
	C7	20 sides (20%)	
Pearson’s correlation coefficient			r
Length of the uncinate process to the size of the adjacent énchancrure			0.8

However, these structures tended to be largest at C3 and C4 vertebral levels and were smallest at C5 and C6 levels. As mentioned above, a C7 énchancrure was uncommon (<20%). The énchancrure was most in contact with the uncinate process with lateral flexion of the cervical spine and in specimens with a longer uncinate process, e.g., C6. The énchancrure was also found to be wider in cases of cervical spine degeneration involving the body of the cervical vertebrae. Degeneration of the uncovertebral joint was most often seen at the énchancrure (approximately 80% of specimens) and not at the adjacent uncinate process.

## Discussion

We found an énchancrure on the majority of sides. The size of these structures was found to have a positive correlation with the length of the uncinate process. The énchancrure was also found to be wider in cases of cervical spine degeneration involving the body of the cervical vertebrae. Degeneration of the uncovertebral joint was most often seen at the énchancrure and not at the adjacent uncinate process. 

The development of the énchancrure and uncinate processes does not take place until early childhood. They are typically seen first between six and nine years of age and do not fully mature until age 18 [1]. The early fissuring in this location leads to the formation of the uncinate process below and the énchancrure above. The fissuring leads to the natural development of a cleft in the lateral margins of the cervical intervertebral disc. This cleft will continue to develop with age, resulting in most adults having more extensive fissuring in the intervertebral disc space between the énchancrure and the uncinate process. These natural changes can, however, become accelerated with injury or disease of the cervical spine [1-4,6]. 

There is controversy as to whether this joint is "real" or due to degenerative processes [1-4,6]. It has been proposed that this joint is the remnant of the complete synovial joint, which is found in lower vertebrates. However, some have considered it to be a true synovial joint [7]. In younger subjects, histology shows loose fibrous and vascular tissues occupying the space between the échancrure and uncinate processes. Moreover, in adults, the cleft that develops from the fissuring has characteristics of a synovial joint [1-3].

Clausen et al. described the function of the énchancrure and uncinate processes as major contributors to coupled motions in the cervical spine. They stated that the énchancrure and uncinate processes function to effectively reduce motion coupling and primary cervical motion in response to axial rotation and lateral bending loads [8,9]. Herniation of the intervertebral disc or compression fractures of the cervical vertebrae can lead to the uncinate process pressing more deeply into the échancrure. Evidence of this was seen in our study, where we found that the énchancrure was wider in cases of cervical spine degeneration involving the body of the cervical vertebrae. This causes an abnormal concentration of forces leading to uncovertebral degeneration with reactive osteophyte formation, causing a protrusion of hypertrophied soft tissue from the medial aspect of the intervertebral foramen [9]. Hypertrophy of the degenerated uncovertebral joint can lead to the narrowing of the intervertebral foramen [8]. 

**Figure 6:**
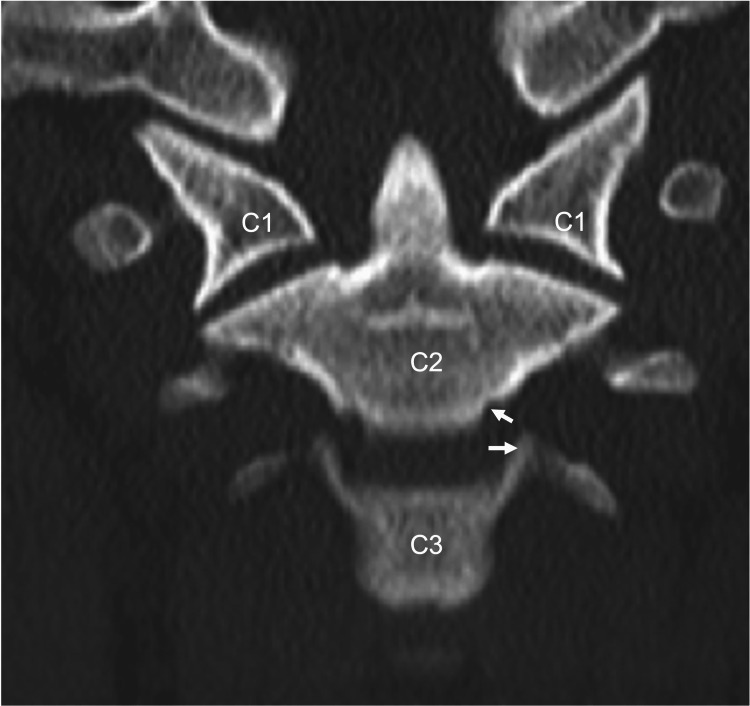
Coronal CT noting the énchancrure (upper arrow) of C2 and uncinate process (lower arrow) of the C3 vertebrae.

A lateral x-ray is imperative for diagnosing fractures of the échancrure. A technique described by Harrison and Macnab demonstrated that manual counter-traction can better disclose énchancrure fractures by optimizing a lateral view [10]. Fractures to the énchancrure or uncinate process can lead to hypermobility of the cervical spine [8]. The énchancrure can also be seen on CT imaging (Figure 6). Narrowing the space between the énchancrure and uncinate processes might help prevent fragments from penetrating laterally during disc herniation [11]. Patients with congenital énchancrure and/or uncinate process absence have an increased risk of lateral disc herniation [12, 13].

Surgically, complete removal of the uncovertebral joint complex during anterior cervical discectomy and fusion procedures is controversial. However, some groups have shown the long-term success of this technique during such procedures in patients with cervical radiculopathy due to hypertrophy of the uncovertebral joint complex [14]. A smaller énchancrure at C5-C6 may contribute to osteophyte formation, which is significantly higher at this level [15]. Furthermore, this structure being uncommonly seen at C7 may explain why osteophyte formation in the inferior zygapophysial joints of this vertebra is significantly higher than that of other cervical vertebrae [16]. As the uncovertebral joint has been found to contribute to cervical spine stability, some authors have proposed only partial removal of the uncinate process during anterior cervical discectomy and fusion procedures. For example, Abudouaini et al. [17] reviewed their patient cohort for such procedures and had good outcomes with such procedures. Others, such as Lee et al. [18], have also minimized the removal of the uncinate process using an oblique resection method. These authors found that such a procedure maintains the stability of the uncovertebral joint but sufficiently decompresses the intervertebral foramen. However, others have not found significant differences between patients undergoing such fusion procedures with or without removal of the uncinate process [19]. Although such procedures have typically focused on the uncinate process of this joint, a better understanding of the interactions between it and the énchancrure could decrease patient morbidity by appreciating the dynamics involved between these two structures, as found in our study.

Limitations

The limitations of this study include not knowing the exact age or sex of the specimens. This restricted us from identifying differences between male and female specimens and elucidating age-related changes in the énchancrure. However, even with these limitations, generalizations about the anatomy of this part of the cervical spine can be made. Lastly, although the specimens were derived primarily from skeletons from North American sources, some varied ethnic differences might not be captured in our study. Future studies that analyze this and age or gender differences are warranted.

Acknowledgment

The authors sincerely thank those who donated their bodies to science so that anatomical research could be performed. Results from such research can potentially increase mankind’s overall knowledge, which can then improve patient care. Therefore, these donors and their families deserve our highest gratitude [20]. 

## Conclusions

We found that the énchancrure is found in the majority of cervical spines. These structures tended to be largest at C3 and C4 vertebral levels and were smallest at C5 and C6 levels, and they had more prominence when the adjacent uncinate process was enlarged. The énchancrure should be considered a normal feature of the inferolateral aspect of the cervical vertebrae. Future clinical studies are necessary to better elucidate their functional significance. 
